# Impacts of Harvesting Activities on the Structure of the Intertidal Macrobenthic Community on Lvhua Island, China

**DOI:** 10.3390/biology14101447

**Published:** 2025-10-20

**Authors:** Shuhan Wang, Yuqing Wang, Jiaming Ou, Jianing Sun, Kaiyi Wang, Qiao Zou, Jianqu Chen, Li Li, Kai Wang, Shouyu Zhang

**Affiliations:** 1 College of Oceanography and Ecological Science, Shanghai Ocean University, Shanghai 201306, China; m230501248@st.shou.edu.cn (S.W.); m17862667869@163.com (Y.W.); 19875921871@163.com (J.O.); 18563055131@163.com (J.S.); m230501249@st.shou.edu.cn (K.W.); z13873939973@163.com (Q.Z.); chenjianqu@scsfri.ac.cn (J.C.); m240300998@st.shou.edu.cn (L.L.); syzhang@shou.edu.cn (S.Z.); 2 Southern Marine Science and Engineering Guangdong Laboratory (Zhuhai), Zhuhai 519082, China; 3 Comprehensive Workstation for Marine Ranching in the East China Sea Region, Expert Consultative Committee on Marine Ranching Construction, Ministry of Agriculture and Rural Affairs, Shanghai 201306, China; 4 Engineering Technology Research Center of Marine Ranching, Shanghai Ocean University, Shanghai 201306, China

**Keywords:** macrobenthos, rocky intertidal zone, biodiversity, functional diversity, remote sensing ecological index

## Abstract

**Simple Summary:**

Understanding the effects of human activities on coastal biodiversity is vital for protecting intertidal ecosystems. On Lvhua Island, many residents harvest shellfish and other marine life, often leading to fewer species and smaller populations in areas near villages. These disturbed sites showed lower ecological stability compared to remote, undisturbed shores, which still supported healthy and diverse communities. Our study shows that frequent harvesting reduces biodiversity and weakens the resilience of the ecosystem. To address this problem, we recommend creating protected zones where harvesting is restricted, giving marine organisms time to recover. Such protection will not only help safeguard the environment but also ensure that fisheries remain a lasting source of food and income for local communities. By balancing conservation with human needs, we can secure both healthy ecosystems and sustainable livelihoods for the future.

**Abstract:**

Human harvesting exerts significant pressure on intertidal ecosystems, yet its impact on community structure remains insufficiently understood. To assess these effects, we investigated macrobenthic communities on Lvhua Island and adjacent islets by integrating ecological surveys, questionnaire data, and Remote Sensing Ecological Indices (RSEI). We analyzed species composition, biomass, density, and diversity indices across seven sampling sites. Results showed distinct spatial variation: the eastern Lvhua Island exhibited higher biomass and density than the west, with the remote Manduishan islet highest and the South of West Lvhua near the pier the lowest. Harvesting hotspots were dominated by *Chlorostoma rusticum* and *Cantharus cecillei*, while less-disturbed islets were characterized by *Chl. rusticum*, *Thais luteostoma*, and Turbinidae. Economically valuable gastropods showed signs of miniaturization under intensive harvesting. Biodiversity indices correlated with RSEI, and ABC curve analysis indicated moderate disturbance overall, with the greatest impact at the Donglvhua Bridge site. These findings indicate that a daily subsistence harvest of 100–150 kg resulted in a 31.82% decline in the Shannon-Wiener index, altering the community structure. RSEI provides a cost-effective complement to field monitoring and should be integrated into management frameworks to support both ecological conservation and community livelihoods.

## 1. Introduction

As a transitional ecological zone between land and sea, the intertidal zone—characterized by dynamic tidal processes—supports highly complex biological communities and harbors rich biodiversity [1,2]. However, this fragile ecosystem is increasingly threatened by both natural and human harvesting stressors. Drastic fluctuations in temperature, salinity, and light intensity make the intertidal zone one of the most sensitive ecosystems in the biosphere [3]. In addition to natural pressures, macrobenthic organisms in intertidal areas are frequently exploited—not only by professionals but also by recreational fishers who collect them as bait [4,5]. Urban expansion, industrial development, and the overexploitation of biological resources have further contributed to habitat degradation and shifts in community structure [6]. In particular, high-intensity development and selective harvesting have led to population miniaturization and age truncation [7]. Specifically, miniaturization refers to the increasing proportion of smaller species in catches [8], while age truncation describes the phenomenon in which the decline of older individuals due to harvesting is substantially greater than that of younger individuals [9]. Case studies demonstrate that miniaturization is particularly evident in overharvested gastropods, and age truncation is not limited to fish but is also significantly observed in invertebrate populations [10,11], such as *Chl. rusticum* from Zhoushan. Furthermore, a limited understanding of the life history and ecological traits of benthic species, along with the absence of reliable data and sustainable exploitation strategies, presents significant challenges for effective resource management [12].

In response to environmental challenges, many countries have expanded the coverage of marine protected areas (MPAs) to achieve a balance between conservation and sustainable use. Among these, Special Marine Protected Areas (SMPAs) emphasize ecosystem integrity while supporting local socio-economic development, thereby offering a model for the sustainable use of marine resources [13,14]. In 2005, China established the Ma’an Archipelago Special Marine Protected Area with the aim of preserving biodiversity and maintaining ecosystem functionality while promoting the rational utilization of resources. Recent domestic research on SMPAs has primarily focused on ecological assessments [15], optimization of management strategies [16], and analysis of benthic community structures [17]. However, the quantification of human harvesting impacts has largely depended on in situ observations or experimental simulations [18,19,20], which are labor-intensive and challenging to scale across extensive areas. These constraints highlight the need for more efficient and scalable assessment tools. In this context, remote sensing technology has emerged as a promising solution, and its role in ecological monitoring and conservation has become increasingly prominent [21,22,23]. Remote sensing-based assessments of human impacts are now commonplace. For instance, Xu [24] proposed the Remote Sensing Ecological Index (RSEI), which integrates greenness, wetness, dryness, and surface temperature, providing a comprehensive, satellite-based metric of ecological quality. RSEI has since been widely applied in various landscape assessments [25], and Liang [26] further demonstrated its potential as a proxy for human disturbance.

In this study, we innovatively apply remote sensing techniques to assess subsistence impacts in the Ma’an Archipelago region, specifically focusing on Lvhua Island and the nearby islets in Zhoushan, Zhejiang Province, China. By combining RSEI analysis with field surveys of intertidal macrobenthic communities—including species composition, biomass, density, and both taxonomic and functional diversity—we aim to provide new insights into the ecological status of intertidal systems under human pressure. The results will serve as valuable references for the planning and management of marine protected areas.

## 2. Materials and Methods

### 2.1. Investigation Area

The Lvhua Island Protected Area (30°49.2′ N, 122°37.8′ E) is situated in Shengsi, Zhoushan City, Zhejiang Province, and is naturally shielded by numerous surrounding islands [27]. The island’s topography features higher elevations in the east and lower elevations in the west. Its coastline is predominantly rocky [28], with gravel beaches primarily located along the central sections of the northern and southern shores [29]. Notably, a mussel aquaculture zone has been established in the southern waters of the island. Positioned at the confluence of the Yangtze River Estuary and the Qiantang River Estuary [30], Lvhua Island exhibits unique hydrological conditions that support a marine ecosystem dominated by intertidal biological communities. Furthermore, designated as a “sustainable use zone” within the Ma’an Archipelago Special Marine Protected Area [31], Lvhua Island features a unique intertidal ecosystem and abundant benthic fauna resources. However, the expansion of mussel aquaculture and intensified human disturbances have placed tremendous pressure on the marine environment of the archipelago, leading to a decline in macrobenthic resources and a reduction in seagrass bed extent [32]. Notably, local residents rely on intertidal biological resources for food and income, and this subsistence harvesting further strains the intertidal environment. Facing the dual pressures of overharvesting and habitat degradation, Lvhua Island provides an ideal setting for researching the impacts of human activities and marine conservation.

### 2.2. Sample Collection

In August and September 2021, the intensity of human disturbance was quantified through a combination of questionnaire surveys and field observations. A questionnaire survey was conducted among local residents on the island (*n* = 20) to collect information related to intertidal harvesting, including harvesting methods, number of harvesters, participants’ ages, harvesting times, primary target species, harvesting frequency, harvest quantities, and the sizes of harvested organisms. Additionally, during a one-week follow-up investigation, gastropod species collected by harvesters were identified and recorded, along with their biomass and the frequency of harvester visits (i.e., the number of independent harvesting events recorded per station during the one-week field observation period).

**Figure 1:**
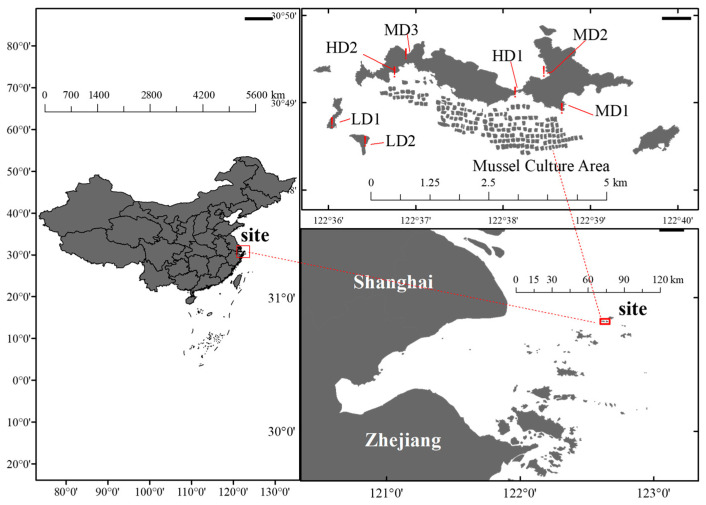
Survey Stations for macrobenthos on Lvhua Island and Its Adjacent Islands.

Seven sampling stations were established on Lvhua Island and its adjacent waters (Figure 1): East Lvhua Bridge (HD1), South of West Lvhua (HD2), South of East Lvhua (MD1), North of East Lvhua (MD2), North of West Lvhua (MD3), Manduishan(LD1), and Mantoushan (LD2). These stations were classified into high-impact zones (HD), moderate-impact zones (MD), and low-impact zones (LD) based on integrated criteria, including substrate type, distance to anthropogenic infrastructure, and the frequency of human harvesting activities. Detailed site characterization data are provided in Supplementary Material Table S1. Human activities varied among the selected sites, including construction and harvesting. For example, HD1 is both a bridge construction site and a location with frequent harvesting activities; HD2 is situated near a pier; MD3, MD2, and MD1 are popular intertidal harvesting sites for local residents; while LD2 and LD1 are remote from the main island, accessible only by boat, with limited harvesting activities.

Three parallel transects were established at each station as spatial replicates (*n* = 3). Following this design, three quadrats (0.25 m × 0.25 m) were sampled per transect—one randomly placed within each of the high, middle, and low intertidal zones—resulting in a total of nine quantitative samples per station. All macrobenthic animals within each quadrat were collected using tools such as shovels and knives. Additionally, qualitative sampling was conducted in the surrounding area of each transect to compile a more comprehensive species inventory for each station. All collected specimens were immediately transported to the laboratory in insulated containers and processed within 24 h. Organisms were identified to the species level based on morphological characteristics using standard taxonomic references, such as The Macrobenthic Assemblages Resources in China’s Coastal Zone, An Illustrated Guide to Species in China’s Seas, and Mollusks of the Yellow Sea and Bohai Sea. Examination under a stereomicroscope was performed when necessary. Key morphological characteristics of dominant taxa were measured, along with their wet weight. All surveys were conducted during spring tides. This timing was strategically chosen for several reasons. First, the larger tidal during spring tides exposed a broader area of the intertidal zone, allowing comprehensive and safe access to the lower intertidal levels. Second, this period coincided with the seasonal fishing moratorium, a time when harvesting pressure on the intertidal zone is intensified, enabling us to better assess its impact on the macrobenthic community. Finally, the weather and sea conditions during these months are typically more stable, ensuring the safety and efficiency of our fieldwork.

### 2.3. Biological Traits Analysis

Based on the characteristics of macrobenthos, this study selected seven functional trait categories—morphology (individual size), life history (lifespan), and behavior (feeding type, trophic type, attachment ability, habitat, and activity)—to describe the functional attributes of macrobenthic communities in Lvhua Island and its adjacent marine areas (Table 1). Trait information for each species was obtained from peer-reviewed journal articles and taxonomic literature [33,34]. This trait information has been used to calculate the following functional diversity indexes: Functional richness (FRic), Functional evenness (FEve), Functional divergence (FDiv), Rao’s quadratic entropy index (RaoQ). Species functional traits were compiled and assigned using a fuzzy coding approach to account for intraspecific variability and uncertainty. Each species was scored from 0 (no affinity) to 1 (full affinity) across various categories within each trait. The complete trait matrix used for analysis is provided in Table S2.

### 2.4. Diversity Indices

In this study, ArcGIS 10.8 was used to generate spatial distribution maps of biomass and biological density, while Origin 2021 was employed to visualize diversity indices. To assess the biodiversity of intertidal macrobenthic invertebrate communities, several ecological indices were applied, including the Shannon-Wiener diversity index (H’), Pielou’s evenness index (E), Margalef’s richness index (d), and Simpson’s diversity index (D). The degree of disturbance to macrobenthic communities was evaluated using ABC (Abundance/Biomass Comparison) curves and the W-statistic.

Relative importance index (IRI) was used to determine species dominance, calculated using the following formula:

(1) $$IRI=\left(N+W\right)\times F\times 10,000$$

*N* is the relative abundance of the species, *W* is the relative biomass of the species, *F* is the frequency of occurrence of the species across all sampling stations, *IRI* value greater than 1000 indicates a dominant species [35].

(2) $$P_{i}=Ni⁄N$$

$P_{i}$ is the proportion of individuals of the i species in the total population. Ni is the number of individuals of species i in the sample, *N* is the total number of individuals of all species in the sample.

(3) $$H^{\prime }=-\sum P_{i}{lnP}_{i}$$

$H^{\prime }$ is the Shannon-Wiener diversity index is the proportion of individuals belonging to species i.

(4) $$E=H^{\prime }/\ln S$$

E represents Pielou’s evenness index, $H^{\prime }$ is the Shannon-Wiener diversity index, S is the total number of species.

(5) d=(s−1)/ln N

d represents Margalef’s richness index, S is the total number of species, *N* is the total number of individuals in the sample.

(6) $$D=1-\sum_{i=1}^{S}p_{i}^{2}$$

*D* represents Simpson’s diversity index, $P_{i}$ is the proportion of individuals belonging to species i.

(7) $$W=\sum_{i=1}^{s}(B_{i}-A_{i})/50(S-1)$$

*W* is the W-statistic used to assess community disturbance, $B_{i}$ is the cumulative biomass proportion of species i, $A_{i}$ is the cumulative abundance proportion of species i, S is the total number of species.

In this study, four functional diversity indices were used to evaluate the functional diversity of macrobenthic communities. FRic represents the total volume of ecological niche space occupied by the community. FEve reflects the regularity of abundance distribution within the occupied niche space. FDiv indicates the extent to which species maximize functional trait differences within the community [36]. RaoQ measures the average functional distance between two randomly selected individuals in the community, integrating both species-level trait differences and their relative abundances [37]. All indices (FRic, FEve, FDiv, and RaoQ) were calculated using the “FD” package in R.

### 2.5. Remote-Sensing Ecological Index

The primary data for this study were obtained from the Landsat 8 OLI_TIRS remote sensing image dataset, which was selected from the Geospatial Data Cloud website (http://www.gscloud.cn/ (accessed on 17 October 2025)). The data were acquired on 28 August 2021, featuring cloud cover of less than 0.2 and exhibiting good image quality. Before calculating the RSEI, the remote sensing images underwent preprocessing using ENVI 5.3, which included radiometric calibration, atmospheric correction, and water body masking. The RSEI values were derived from the normalization of four remote sensing indices and principal component analysis. RSEI was calculated using the following formula [38]:

(8) $$RSEI=f\left(Wet,\;NDVI,\;LST,\;NDBSI\right)$$

WET represents Wetness, Wetness indicates the moisture content in surface vegetation and soil on Lvhua Island and is a key metric for assessing ecological quality, commonly extracted using the Kirchhoff Transform.; NDVI represents greenness, Greenness primarily reflects plant growth status and coverage in a region, typically measured and normalized via vegetation indices.; LST is represented by the Land Surface Temperature of Lvhua Island, generally derived from the thermal infrared band of Landsat imagery; and NDBSI represents dryness, Dryness is characterized by the normalized difference build-up and the soil Index, which synthesizes built-up and bare soil indices to analyze soil erosion conditions in the area. The detailed calculation formulas are provided in the Supplementary Material (Formulas).

### 2.6. Data Analysis

All statistical analyses were performed using R software (version 4.4.3). Prior to statistical testing, all data were checked for assumptions of normality and homogeneity of variances using the Shapiro-Wilk test and Levene’s test, respectively. For data that violated these assumptions, appropriate transformations were applied, or non-parametric tests were used.

To assess the differences in environmental variables and univariate community descriptors among the three disturbance zones (HD, MD, LD) and three tidal levels (High, Middle, Low), a two-way analysis of variance (ANOVA) was employed, followed by Tukey’s Honest Significant Difference (HSD) post-hoc test for multiple comparisons. When the assumptions for ANOVA were not met even after transformation, the non-parametric Kruskal-Wallis test was used, followed by Dunn’s test for post-hoc analysis. All statistical tests were considered significant at a confidence level of α = 0.05.

The relationship between RSEI and biodiversity was analyzed using Spearman’s rank correlation coefficient. The Spearman correlation analysis was performed using Origin(version 2024; OriginLab Corporation, Northampton, MA, USA).

## 3. Results

### 3.1. Questionnaire Survey and Field Observations

The results of the 2021 questionnaire survey (*n* = 20) indicate that intertidal harvesting on Lvhua Island represents a typical subsistence fishery. Over 95% of households rely on high-value gastropods such as *T. luteostoma*, *Chl. rusticum*, Turbinidae and *Monodonta labio* as sources of both food and income, with some households depending on them as their sole economic livelihood. During the one-week field observation, harvesting activities were concentrated on spring tide days at the lowest tide points, occurring twice daily. The primary harvesting method was manual collection, assisted by simple tools, with approximately 5–6 harvesters observed per station per event, mainly targeting gastropods. Harvesting activities are concentrated during spring tide periods, with an average individual harvest of 3.5 ± 1.2 kg per spring tide cycle. Specifically, the average harvest amounts for *T. luteostoma*, *Chl. rusticum*, Turbinidae and *M. labio* were 1.5 ± 1.1 kg, 1.8 ± 1.1 kg, 2.4 ± 1.8 kg, and 0.8 ± 0.7 kg, respectively. Based on a questionnaire survey and field investigations conducted among 20 households, the average per capita harvest was determined to be 3.5 kg per day. Since not all household members participate in harvesting activities, the number of active harvesters was estimated to be between 20 and 40 individuals. Accordingly, the total daily harvest during spring tide periods was estimated to range from 70 to 140 kg.

Analysis of gastropods collected by residents revealed a harvesting bias favoring larger individuals compared to population averages obtained from quantitative quadrats. Statistical comparisons were performed among three defined groups: (1) manually collected specimens, (2) individuals from general quantitative quadrats, and (3) individuals from low-impact zones.

For *Chl. rusticum*, Kruskal-Wallis H tests revealed significant differences among the three groups (total *N* = 1636). Regarding shell length (H = 61.790, df = 2, *p* < 0.001), post hoc Dunn’s tests with Bonferroni correction indicated that manually collected individuals (21.44 ± 3.50 mm) were significantly larger than those from general quadrats (19.18 ± 5.56 mm; *p* < 0.01). Furthermore, specimens from low-impact zones (23.43 ± 2.79 mm) were significantly larger than manually collected individuals (*p* = 0.013). Regarding body weight (H = 26.735, df = 2, *p* < 0.001), post-hoc analysis showed that manually collected individuals (4.66 ± 1.83 g) were significantly heavier than those from general quadrats (4.07 ± 2.15 g; *p* < 0.01). However, no significant difference was found between the body weight of manually collected individuals and those from low-impact zones (6.40 ± 2.69 g; *p* = 0.315).

For *T. luteostoma*, significant differences were also observed among the three groups (total *N* = 670). Regarding shell length (H = 7.509, df = 2, *p* = 0.023), post-hoc tests revealed that manually collected individuals (35.50 ± 5.85 mm) were significantly larger than those from general quadrats (34.12 ± 7.74 mm; *p* = 0.029). However, the shell length of individuals from low-impact zones (34.99 ± 7.78 mm) did not differ significantly from that of manually collected specimens (*p* > 0.05). For body weight (H = 18.207, df = 2, *p* < 0.001), manually collected individuals (8.57 ± 2.46 g) were significantly heavier than those from general quadrats (5.68 ± 3.55 g; *p* < 0.01). No significant difference was observed between the body weight of manually collected individuals and those from low-impact zones (7.80 ± 3.02 g; *p* = 0.901).

For *Anthocidaris crassispina*, the average test diameter and body weight of manually collected individuals were 55.59 ± 14.33 mm and 87.62 ± 22.52 g, respectively. These measurements represent the size range of individuals targeted by harvesters. A formal statistical comparison with quadrat samples was not conducted due to an insufficient number of *A. crassispina* encountered within the quadrats.

In terms of spatial distribution, data from both the questionnaire and field observations indicated that HD1, MD1, and MD2 were the primary hotspots for harvesting activity. A small number of residents also traveled by boat to LD2 and LD1 to collect gastropods. Observational data recorded the number of harvesting events as follows: MD2 (14 frequency, 22.95%), MD1 (15 frequency, 24.59%), HD1 (12 frequency, 19.67%), MD3 and HD2 combined (8 frequency, 13.11%), and LD2 (4 frequency, 6.56%). The age structure of harvest participants exhibited a significant aging trend, with individuals aged 55 and older accounting for 80% of the total.

### 3.2. Species Composition and Dominant Species

A total of 29 macrobenthic species were identified across the seven sampling stations on Lvhua Island and its adjacent islets, a complete checklist of the 29 macrobenthic species identified in this study, including their taxonomy and distribution across sampling sites, is provided in the Supplementary Material (Table S3). Among these, Mollusca was the most dominant phylum, comprising 21 species (72.41%). Echinodermata and Cnidaria each accounted for 2 species (6.90%), while Arthropoda included 4 species (13.79%). Collectively, the non-molluscan taxa contributed 27.59% to the total species richness. The LD1 station exhibited the highest species richness, with 21 species recorded, followed by HD1 (15 species), MD1 (12 species), LD2 (11 species), MD3 (10 species), and HD2 (9 species). The MD2 station had the lowest species richness, with only 7 species identified.

The dominant species varied across sites (Table 2). *T. clavigera* was dominant at MD1, MD2, MD3, and HD2. *Can. Cecillei* was dominant at MD1, MD2, HD2, and LD1. *T. clavigera* was dominant at MD2, LD1, and LD2. *Chl. rusticum* was dominant at MD2, MD3, and HD1. *Tetraclita japonica* was dominant at MD3 and HD2. *Turbo spp.* and *T. luteostoma* were the least frequently observed dominant species, occurring primarily at LD1 and LD2, respectively. Notably, MD2 had the highest number of dominant species, while HD1 exhibited the fewest.

### 3.3. Biomass and Biological Density

**Figure 2:**
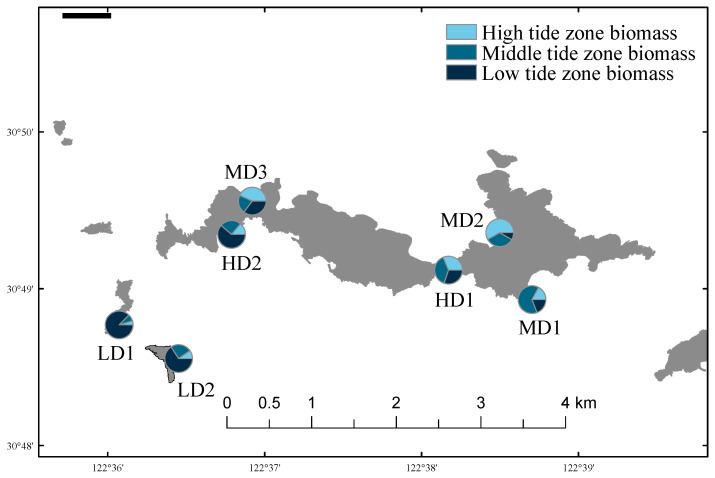
Spatial distribution of biomass in Lvhua Island and adjacent islands.

**Figure 3:**
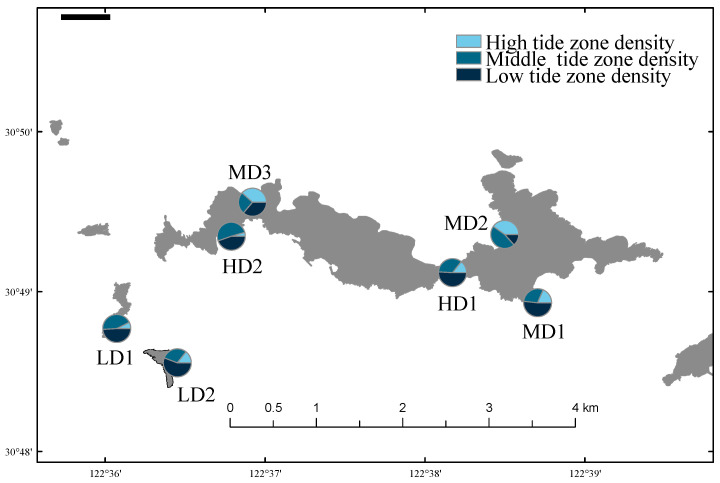
Spatial distribution of density in Lvhua Island and adjacent islands.

The variations in macrobenthic biomass and density across the seven sampling stations on Lvhua Island and its adjacent islets are presented in Figure 2 and Figure 3. The average biomass of benthic organisms ranged from 83.24 ± 27.49 g/m^2^ to 907.90 ± 1276.89 g/m^2^ (*n* = 9), while the average density ranged from 16.71 ± 3.43 ind./m^2^ to 94.22 ± 40.12 ind./m^2^ (*n* = 9). The LD1 station exhibited the highest values for both biomass and density, which were approximately 10.9 times and 4.4 times higher than those recorded at the MD3 station, respectively. Peak values were consistently observed in the low intertidal zone at LD1. Although differences in biomass and density were observed, the Kruskal-Wallis tests indicated no statistically significant differences in macrobenthic biomass (H = 6.814, df = 6, *p* = 0.338) or biological density (H = 9.177, df = 6, *p* = 0.164) among the different disturbance zones.

In terms of vertical distribution, the biomass and density at LD1, LD2, and MD1 generally followed the typical intertidal pattern of low tide zone > mid tide zone > high tide zone. However, an abnormal distribution was observed at MD3, where the order was high > low > mid tide zone. Additionally, at HD1, the biomass peak shifted to the mid tide zone, deviating from the expected vertical trend.

### 3.4. Biodiversity Indices and ABC Curves

**Figure 4:**
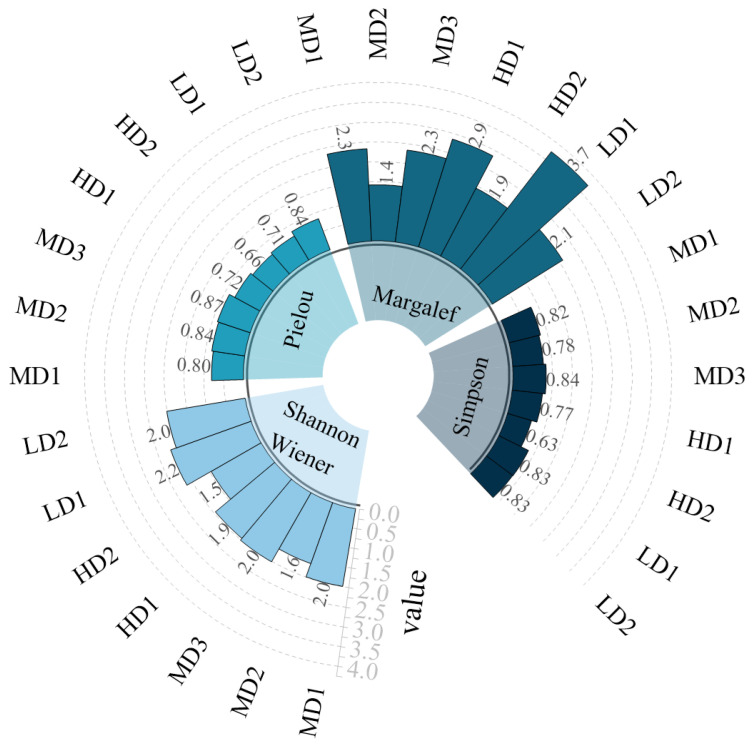
Biodiversity Indices of Macrozoobenthos on Lvhua Island and Adjacent.

Biodiversity indices varied among the sampling stations (Figure 4). The highest Shannon–Wiener index was recorded at LD1 (2.2), while the lowest was observed at HD2 (1.5). Margalef’s richness index also peaked at LD1 (3.7) and reached its lowest value at MD2 (1.3). In contrast, Simpson’s dominance index and Pielou’s evenness index exhibited relatively minor variation across the stations.

**Figure 5:**
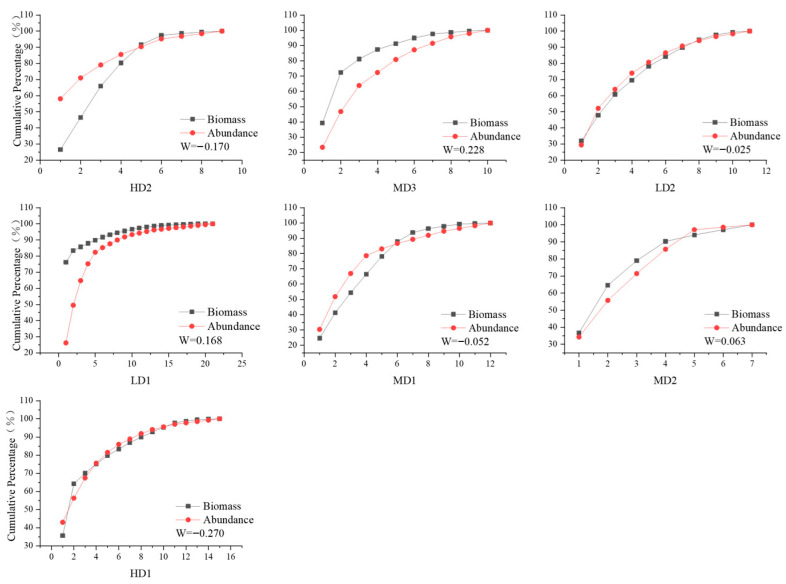
ABC Curve of Macrozoobenthos Communities on Lvhua Island and Adjacent Islands.

According to the Abundance–Biomass Comparison (ABC) curves (Figure 5), five stations—MD1, HD2, HD1, LD2, and MD2—exhibited curve crossings or overlaps, indicating disturbed community structures. Among these, MD1, HD2, HD1, and LD2 had negative W values, suggesting high levels of anthropogenic disturbance and ecological instability. In contrast, the LD1 and MD3 stations displayed biomass curves consistently above the abundance curves, with no crossover; their corresponding W values were 0.168 and 0.228, respectively. The HD1 station had the most negative W value (−0.270), indicating the highest degree of disturbance among all stations.

**Figure 6:**
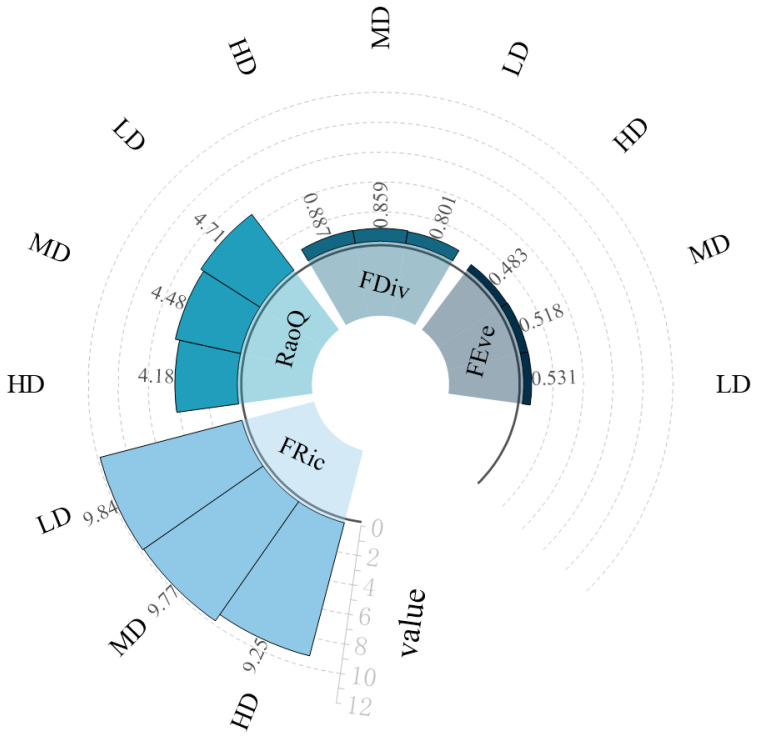
Functional Diversity Indices of Macrozoobenthos Communities on Lvhua Island and Adjacent Islands. Note: HD (High-impact zones: HD1, HD2); MD (Moderate-impact zones: MD1, MD2, and MD3); LD (Low-impact zones: LD1 and LD2).

Functional diversity was assessed across these zones. Functional diversity of macrobenthic communities was calculated for the three regions, based on the biological trait matrix provided in Supplementary File S1. As illustrated in Figure 6, the indices FRi, FEve, and RaoQ were highest in the low-impact areas and lowest in the high-impact areas. Conversely, the index FDiv was highest in the high-impact areas and lowest in the low-impact areas.

### 3.5. Remote Sensing Ecological Index

**Figure 7:**
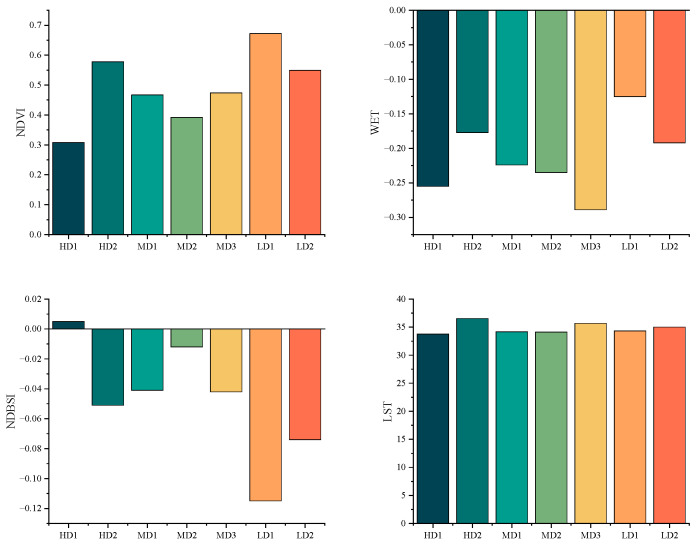
Average Remote Sensing Index at Each Station.

**Figure 8:**
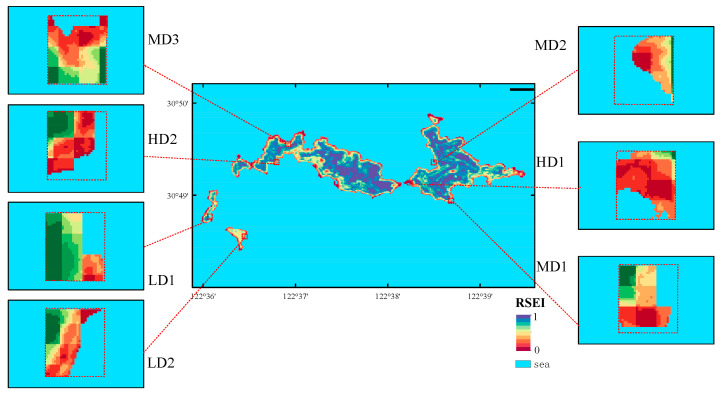
Spatial distribution of the Remote Sensing Ecological Index (RSEI) values at each sampling station. Note: The area of each red rectangle is 100 m^2^.

The average NDVI value at the LD1 site was 0.672, the highest among all sites, while the HD1 site recorded the lowest average value at 0.308. The WET index peaked at the LD1 site with an average value of -0.125, whereas the lowest average value was observed at the LD2 site (−0.289). The MD3 site exhibited the highest average LST value at 36 °C, while the HD1 site had the lowest at 34 °C. Additionally, the HD1 site demonstrated the highest average NDBSI value (0.005), whereas the LD1 site had the lowest (−0.115) (Figure 7). Finally, the highest RSEI value was recorded at LD1 (0.646), and the lowest at HD1 (0.187) (Figure 8).

### 3.6. Relationships Between Macrobenthic Biodiversity, RSEI, Biomass, and Density

**Figure 9:**
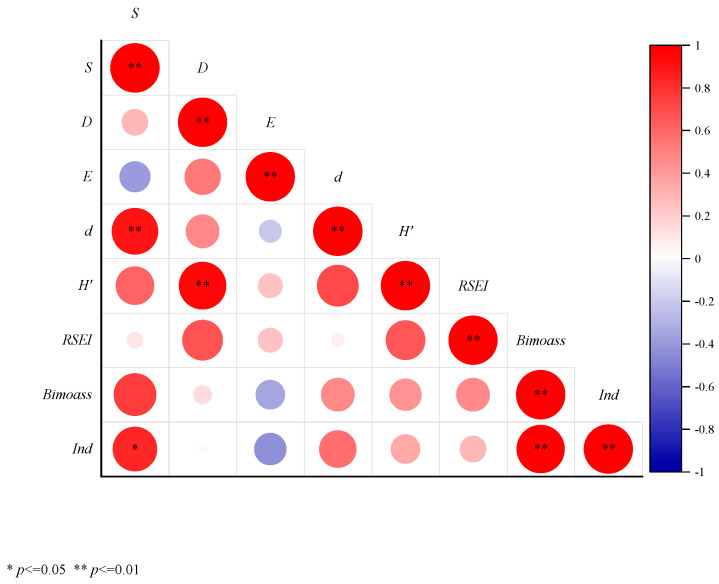
Results of Spearman Correlation Analysis. Note: S: Species Richness, D: Simpson Diversity Index, E: Evenness Index, d: Richness Index, H’: Shannon-Wiener Index, RSEI: Remote Sensing Ecological Index, Biomass: Biomass, Ind: Biological Abundance.

In the Spearman correlation analysis (Figure 9), S is positively correlated with H’, RSEI, and Biomass, while it is negatively correlated with E. S shows a significant positive correlation with d and Ind. D is positively correlated with E, d, RSEI, and Biomass, and it is significantly positively correlated with H’. E is positively correlated with H’ but negatively correlated with d, RSEI, Biomass, and Ind. d is positively correlated with H’, RSEI, Biomass, and Ind. H’ is positively correlated with RSEI, Biomass, and Ind. RSEI is positively correlated with Biomass and Ind. Finally, Biomass is significantly positively correlated with Ind.

## 4. Discussion

This study aimed to assess the impacts of human harvesting activities on the structure of the intertidal macrobenthic community on Lvhua Island by integrating ecological surveys, questionnaire data, and Remote Sensing Ecological Index (RSEI) analysis. Specifically, we sought to: (1) quantify the spatial variation in species composition, biomass, density, and biodiversity indices; (2) evaluate the relationship between macrobenthic community structure and anthropogenic disturbance using ABC curves and functional diversity indices; and (3) explore the utility of RSEI as a cost-effective tool for assessing ecological quality in the intertidal zone. Our findings provide new insights into these objectives.

### 4.1. Regional Differences in Macrobenthic Communities

A total of 29 macrobenthic species were collected in this study, with Mollusca being the most dominant phylum, comprising 21 species (72.41% of the total). In comparison to previous studies, the number of species recorded in this survey was notably lower than those reported from Zhoushan Island (36 species), the Nanji Archipelago (108 species), and uninhabited islands (58 species) [39,40,41]. This discrepancy may be attributed to habitat conditions. Lvhua Island features a typical erosional coast characterized by strong wave action [42], which may have contributed to the relatively low species richness of macrobenthos. This study found that the species composition of Lvhua Island was similar to that of other intertidal investigations, with Mollusca maintaining a dominant presence [43]. There were significant differences in species numbers among stations, with LD1 recording the highest number of species and MD2 the lowest. At the HD2 station, where port facilities were observed, marine development activities may have directly or indirectly impacted the intertidal habitat. Such activities are known to degrade benthic habitats and reduce macrobenthic species richness [44], which is consistent with the lower number of species we recorded at this site. MD2 site is subject to significant human activity, with a relatively high harvesting frequency (22.95%), which may have contributed to the reduction in species richness at this site.

The composition of dominant species serves as a crucial indicator of community structure and habitat characteristics. In this study, the dominant species with high relative importance indices were predominantly mollusks, aligning with findings from previous research on rocky intertidal zones [45,46]. However, a horizontal comparison of the dominant species between remote stations (LD1, LD2) and those on the main island revealed a significant reduction or even absence of economically important species such as *Turbo* spp. and *T. luteostoma* on the main island. On one hand, these economically valuable gastropods provide essential livelihoods for coastal communities; on the other hand, long-term, high-intensity selective harvesting has resulted in the loss of key species. For example, the average harvest can reach 70 kg per person per day, with the primary target species being *T. luteostoma* and *Chl. rusticum*, potentially triggering trophic cascades and ecosystem simplification [47], for example, *T. luteostoma* is a carnivorous predator that helps control the populations of its prey, while *Chl. rusticum* is a herbivorous grazer that plays a critical role in controlling algal growth on rocky surfaces. Consequently, overharvesting may disrupt both top-down and bottom-up ecological processes in the intertidal ecosystem. Human harvesting not only directly diminishes the population size of economically important species [48] but also contributes to local habitat fragmentation through activities such as trampling and rock-turning [49], depriving species of the necessary conditions and resources for survival.

The LD1 station recorded the highest biomass and density, which were 10.9 times and 4.4 times greater than those of the HD2 station, which exhibited the lowest biomass. The biomass observed in this study was significantly lower than previous findings. A 2006 survey conducted on Zhoushan Island reported an average biomass of 5867.18 g/m^2^ [38]; a 2012 survey in the Nanji Archipelago recorded 3748.6 g/m^2^ [39]; and a 2019 survey of uninhabited islands documented 2847.83 g/m^2^ in rocky intertidal zones [40]. Biomass and density were higher in eastern Lvhua compared to western Lvhua, greater in the south than in the north, and significantly elevated on adjacent islets compared to Lvhua Island. The increased biomass in eastern Lvhua may be attributed to its open degree of seashore, which promotes the growth of sessile and soft-bodied organisms [50].

In terms of vertical distribution, the overall pattern observed is high tide zone < mid tide zone < low tide zone, which aligns with previous findings from intertidal investigations in the Ma’an Archipelago [50]. The high tide zone is exposed to air for long periods, necessitating that organisms tolerate extreme temperature fluctuations and ultraviolet radiation [51]. Benthic animals in this zone often suffer from water loss due to evaporation, and only a few species, such as *T. japonica*, possess physiological adaptations to resist desiccation. Most benthic species cannot endure the dry and UV-intensive environment of the high tide zone [52], resulting in significantly lower biomass and density compared to the mid and low tide zones. Conversely, the low tide zone, which remains submerged for longer periods, provides a more stable environment that supports widespread algal growth, providing suitable habitats for benthic fauna [53]. At the MD3 station in western Lvhua, an unusual distribution pattern was observed, with biomass and density following the sequence of high tide > low tide > mid tide. This phenomenon may be attributed to the relatively flat terrain at this site [54], which facilitates local residents in conducting harvesting activities. Such harvesting practices typically result in reductions in both species richness and abundance [55].

### 4.2. Analysis of Community Structure Stability and Its Response to RSEI

The stability of macrobenthic community structure is determined by multiple factors and can be assessed using diversity indices and ABC curves to evaluate the level of disturbance and community stability [56,57]. In this study, the ABC curve was combined with the RSEI to quantitatively assess the impact of human activities.

The results showed a significant positive correlation between the Shannon–Wiener index and the Simpson diversity index. This indicates that areas with higher Shannon–Wiener index values tend to have more evenly distributed species and a lower proportion of dominant species, reflecting a more stable community structure. For example, the LD1 station exhibited the highest Shannon–Wiener index and a stable ABC curve, suggesting that the community in this low-disturbance area is more intact. In contrast, the HD2 station had the lowest diversity indices, and its biomass and abundance curves intersected, indicating a relatively unstable community structure [58]. The harvesting of larger gastropod individuals reduces the disparity between abundance-based and biomass-based dominance, resulting in the convergence or partial overlap of the biomass and abundance curves. At the LD2 and LD1 stations, lower harvesting frequencies help avoid targeted collection of economically valuable gastropods. The ABC curves confirmed that communities in these areas have not been subjected to significant environmental disturbance, whereas both the eastern and western Lvhua stations exhibited signs of more severe impacts.

Analysis incorporating the RSEI indicated that the average RSEI values in this study were comparable to those observed in urban areas [59], demonstrates that the application of the RSEI to intertidal zones is feasible. The HD1 station had the lowest RSEI value, exhibiting a lower NDVI than other stations and a higher NDBSI, likely due to the impact of bridge construction. Construction debris may have altered habitats previously suitable for gastropod mollusks [60]. In contrast, the LD1 station had the highest RSEI value, characterized by a higher NDVI and a lower NDBSI compared to other stations. The geographical isolation of this site from the mainland reduces the intensity of human activity and supports more intact vegetation cover. The synergistic effect of these factors creates a more stable and less stressed environment, as reflected by its RSEI value. Previous studies have shown that human activities negatively affect the environment and lead to decreased RSEI values [61,62]. Furthermore, our correlation analysis confirms a positive relationship between the RSEI and species diversity indices. This finding elucidates the pathway through which human activities significantly impact the stability of intertidal macrobenthic communities. For instance, harvesting activities often involve turning stones and trampling, which directly damage vegetation integrity and lead to habitat degradation. Similarly, the construction of infrastructure, such as piers and bridges, reduces NDVI and increases the NDBSI index [26], reflecting a decline in vegetation and a rise in built-up/barren areas. Consequently, our findings demonstrate that human activities primarily alter habitat conditions, as visually captured by a decrease in the RSEI. This habitat degradation subsequently erodes biodiversity, which in turn destabilizes the structure of the intertidal macrobenthic community.

### 4.3. Functional Diversity Analysis of Macrobenthic Fauna

In the low-impact zone, FRic, FEve, and RaoQ values were relatively high, indicating that ecological niches were fully occupied, community resources were efficiently utilized, and the ecosystem exhibited higher stability [63]. FRic was significantly positively correlated with species abundance and species richness [37], which corresponds to the observed results—higher FRic values and a relatively more macrobenthic species in the low-impact zone.

Lower FEve values are usually attributed to homogeneous biological traits [64,65]. For example, 16 macrobenthic species were collected from the high-impact stations HD1 and HD2, among which *Chl. rusticum* accounted for 31% of the species composition. This trait homogenization contributed to the lower FEve observed in high-impact areas.

RaoQ incorporates both FRic and FEve components of functional diversity and better reflects the community’s ability to maintain functional diversity under stable conditions. The results indicated that the low-impact zone exhibited higher stability compared to the high-impact zones.

FDiv characterizes niche complementarity among species within a community. Higher FDiv values indicate lower niche overlap and reduced resource competition, meaning more efficient resource use. Compared to other regional studies [65,66], the FDiv values in this study ranged from 0.801 to 0.887, which is still considered high. All impact zones (high, moderate, and low) exhibited high FDiv values, suggesting a high degree of niche differentiation and low interspecific competition [67].

In this study, a large number of species with distinct trait combinations was observed. For example, in the low-impact station LD1, the first dominant species was *Chl. rusticum*, a herbivorous grazer; the second dominant species was *Turbo* spp., a carnivorous predator; and the third dominant species was *Can. cecillei*, a detritivore that primarily collects organic particles.

### 4.4. Impacts of Harvesting on the Intertidal Zone

Harvesting activities on Lvhua Island primarily occur in the intertidal zone during low tide and represent a typical subsistence fishery. Notably, 80% of the harvesters belong to the older age group. Their harvesting behavior is highly selective, mainly targeting large-sized, economically valuable gastropods. Moreover, coastal harvesting is a long-term, continuous activity and constitutes an integral part of the livelihoods of island residents [20,68]. Human harvesting activities lead to habitat fragmentation, and the selective removal of resources reduces the stability of large benthic communities [69]. The sampling stations on the main island already exhibit an imbalance in the abundance of key species. This is likely due to persistent human harvesting activities, which remove large, dominant individuals and consequently alter the size structure of the exploited populations. Existing research indicates that such alterations to population size structure can have detrimental effects on exploited populations [70]. Although the stations on nearby islands have not yet been subjected to intense disturbance, their ABC curves reveal a trend of convergence between the biomass and density lines, highlighting the need for increased conservation attention for these adjacent islands.

A cross-site comparison of phenotypic traits of *Chl. rusticum* and *T. luteostoma* shows that individuals from LD1 and LD2—stations located at a distance from the main island—have significantly larger shell lengths and widths than those from other sites. These offshore reefs are separated from the main island and can only be accessed by boat. Numerous studies have indicated that coastal limited accessibility is a key factor in reducing harvesting pressure on intertidal gastropods [20,71]. Globally, in areas where mollusk collectors are active, a reduction in shell length of harvested species is common [19,72,73]. One study even found that only six weeks of manual harvesting could reduce gastropod shell length from 130 mm to 107.61 mm [74].

Historical records indicate that the maximum shell length of *Chl. rusticum* in the Zhoushan area is 32.40 mm, with common sizes ranging from 25 to 30 mm [7]. In this study, individuals from the LD1 and LD2 stations exhibited phenotypic traits highly consistent with historical records. In contrast, individuals from sites adjacent to human activity zones showed significantly smaller sizes, reflecting the influence of subsistence harvesting on *Chl. rusticum*. In addition to the overall decline in phenotypic traits, selective harvesting by fishers may also affect population sex ratios. Many studies [75,76,77] have demonstrated that manual harvesting tends to preferentially remove larger individuals, which are often females. This disrupts the sex ratio of populations, reduces recruitment rates, and may even lead to local extinction of species [78].

## 5. Conclusions

A total of 29 benthic species from four phyla were identified across seven sampling stations on Lvhua Island and its adjacent island. The LD1 station recorded the highest biomass and density, while the MD3 station showed the lowest. The LD1 station had the highest Shannon–Wiener index, richness index, and RSEI value. In contrast, the HD1 station exhibited the lowest RSEI value and a marked decline in biodiversity indices. ABC curve analysis further revealed that the biomass and abundance curves at HD1 overlapped and intersected, resulting in a negative W value, which indicates poor community stability. In comparison, the community structure at the LD1 station was more stable.

To investigate the ecological gradient of human disturbance, the seven stations were selected a priori and classified into GYX (HD1, HD2), ZYX (MD1, MD2, MD3), and DYX (LD1, LD2). Consistent with this classification, the high-impact zones exhibited the lowest values of FRic, FEve, and RaoQ, reflecting the least stable community structures.

Excessive coastal harvesting by humans has led to a decline of economically valuable gastropod species, while smaller, non-edible species have gained dominance within these communities. This shift may undermine the long-term sustainability of benthic resources. Additionally, frequent coastal harvesting contributes to habitat fragmentation, further increasing the vulnerability of intertidal ecosystems.

Based on our findings, we propose designating the high-biodiversity, low-disturbance sites LD1 (RSEI = 0.646) and LD2 (RSEI = 0.507) as core no-take zones, prohibiting all harvesting activities. For severely degraded areas such as HD1 (RSEI = 0.187), we recommend active restoration measures, including habitat cleanup and transplantation of key species. In moderately disturbed zones (e.g., HD2, MD2), we suggest implementing community co-management plans that incorporate size limits and seasonal closures, supported by integrated RSEI assessments and field monitoring. The RSEI can serve as an indirect indicator for evaluating intertidal ecosystem conditions and holds significant potential for further development in coastal interface zones. By integrating field surveys with remote sensing data, coastal resource management can be significantly improved. Additionally, the establishment of no-take zones is recommended to protect benthic resources.

## Figures and Tables

**Table 1:** Biological Traits of macrobenthos.

Biological Traits	Trait Categories	Labels
Individual Size	<1 cm	Size1
	1–3 cm	Size2
	3–5 cm	Size3
	>5 cm	Size4
Lifespan	Long	L1
	Medium	L2
	Short	L3
Feeding type	Car	X1
	Herb	X2
	Omni	X3
	Detr	X4
Feeding groups	Predator	Feed1
	Scrapers	Feed2
	Filter-collectors	Feed3
	Gather-collectors	Feed4
Attachment ability	Yes	Y1
	No	Y2
	Both	Y3
Habitat	Attach	H1
	Crawl	H2
	Burrow	H3
Activity	Sessile	A1
	Semi-motile	A2
	Motile	A3

**Table 2:** Dominant Species Composition of Lvhua Island and the Neighboring Islands.

Site	Phylum–Order	Dominant Species	IRI
MD1	Mollusca–Neogastropoda	*Cantharus cecillei*	2401.63
	Mollusca–Neogastropoda	*Thais clavigera*	1206.07
MD2	Mollusca–Archaeogastropoda	*Chlorostoma rusticum*	3381.50
	Mollusca–Neogastropoda	*Cantharus cecillei*	1870.09
	Arthropoda–Sessilia	*Tetraclita japonica*	1405.96
	Mollusca–Neogastropoda	*Thais clavigera*	1076.06
MD3	Mesogastropoda–Serpulorbis	*Serpulorbis imbricata*	2047.14
	Arthropoda–Sessilia	*Tetraclita japonica*	1886.54
	Mollusca–Neogastropoda	*Thais clavigera*	1187.18
HD1	Arthropoda–Sessilia	*Tetraclita japonica*	2617.53
HD2	Mollusca–Neogastropoda	*Thais clavigera*	4456.97
	Mesogastropoda–Serpulorbis	*Serpulorbis imbricata*	1344.89
	Mollusca–Neogastropoda	*Cantharus cecillei*	1283.63
LD1	Mollusca–Archaeogastropoda	*Chlorostoma rusticum*	1449.25
	Mollusca–Neogastropoda	Turbinidae	1463.42
	Mollusca–Neogastropoda	*Cantharus cecillei*	1006.74
LD2	Mollusca–Archaeogastropoda	*Chlorostoma rusticum*	2018.17
	Mollusca–Neogastropoda	*Thais luteostoma*	1287.27

## Data Availability

The data that support the findings of this study are available from the corresponding author, [Wang Kai], upon reasonable request.

## Supplementary Materials

The following supporting information can be downloaded at: https://www.mdpi.com/article/10.3390/biology14101447/s1, Table S1: Site characterization of the intertidal sampling stations on Lvhua Island; Table S2: Functional Traits for Macrobenthic Species; Table S3: The Species Composition of the Macrobenthic Community at Lühua Island; File S1: Supplementary Material (Formulas).
