# Supplementary material for: Impacts of Harvesting Activities on the Structure of the Intertidal Macrobenthic Community on Lvhua Island, China
Source: Biology (Basel). 2025 Oct 20;14(10):1447. doi: 10.3390/biology14101447 (PMC12561447; doi:10.3390/biology14101447)
Supplement: Supplementary file 1 [file biology-14-01447-s001.zip › File S1:supplementary material(Formulas).pdf]

The greenness index is represented by the Normalized Difference Vegetation Index (NDVI), which reflects the state of plant growth, vegetation density distribution, and vegetation coverage.

$$NDVI = (\rho_{NIR} - \rho_{Red})/(\rho_{NIR} + \rho_{Red}) \quad (9)$$

ρ_{NIR}—near-infrared band. ρ_{Red}—red band

The humidity component is calculated using the cap transformation, which reflects the surface water conditions.

$$WET_{OLI} = 0.3283\rho_{Red} + 0.1972\rho_{Green} + 0.1511\rho_{Blue} + 0.3407\rho_{NIR} - 0.7117\rho_{SWIRl} - 0.4559\rho_{SWIR2} \quad (10)$$

ρ_{Red} — red band. ρ_{Green} —green band. ρ_{Blue} —blue band. ρ_{NIR} —near-infrared band. ρ_{SWIR1}— the shortwave infrared band 1. ρ_{SWIR2}—the shortwave infrared band 2

The dryness is calculated using the average value of the IBI (Building Index) and SI (Bare Soil Index), as follows:

$$IBI = (2\rho_{SWIR}/(\rho_{SWIRl} + \rho_{NIR}) - \rho_{NIR}/(\rho_{Red} + \rho_{NIR}) - \rho_{Green}/(\rho_{Green} + \rho_{SWIRl})) \,/\, (2\rho_{SWIRl}/(\rho_{SWIRl} + \rho_{NIR}) + \rho_{NIR}/(\rho_{Red} + \rho_{NIR}) + \rho_{Green}/(\rho_{Green} + \rho_{SWIRl})) \quad (11)$$

$$SI = [(\rho_{SWIRl} + \rho_{Red}) - (\rho_{Blue} + \rho_{NIR})]/[(\rho_{SWIRl} + \rho_{Red}) + (\rho_{Blue} + \rho_{NIR})] \quad (12)$$

$$NDBSI = (IBI + SI)/2 \quad (13)$$

ρ_{Red} — red band. ρ_{Green} —green band. ρ_{Blue} —blue band. ρ_{NIR} —near-infrared band. ρ_{SWIR1}— the shortwave infrared band 1. ρ_{SWIR2}—the shortwave infrared band 2

The land surface temperature, which represents the thermal index, can be calculated using the model from the Landsat User Manual and the latest calibration parameters revised by Chander.

$$L = gain \times \mathrm{DN} + \mathrm{bias} \quad (14)$$

$$T = K_2/ln\,(K_1/L + 1) \quad (15)$$

$$LST = T/(1 + (\lambda T/\alpha)\ ln\,\varepsilon) - 273.15 \quad (16)$$

L—the radiance value of the Landsat 8 . *DN*—gray level. *gain*—the band gain. *bias*—the band offset,. *T*—the sensor temperature. K_1K_2—calibration parameters. λ—central wavelength of the thermal infrared band. α=1.438×10^{-2}/m·k. ε—he surface emissivity.
