# Supplementary material for: Impacts of Harvesting Activities on the Structure of the Intertidal Macrobenthic Community on Lvhua Island, China
Source: Biology (Basel). 2025 Oct 20;14(10):1447. doi: 10.3390/biology14101447 (PMC12561447; doi:10.3390/biology14101447)
Supplement: Supplementary file 1 [file biology-14-01447-s001.zip › Supplementary Material (Table S3).pdf]

TableS3:The Species Composition of the Macrobenthic Community at Lühua Island

Phylum	Class	Order	Family	Genus	Species	MD1	MD2	MD3	HD1	HD2	LD1	LD2
Arthropoda	Crustacea	Sessilia	Balanidae	Balanus	*Balanus trigonus*				+			
Arthropoda	Crustacea	Sessilia	Tetraclitidae	Tetraclita	*Tetraclita japonica*	+	+	+	+	+	+	
Arthropoda	Crustacea	Sessilia	Tetraclitidae	Tetraclita	*Tetraclita squamosa squamosa*				+			
Arthropoda	Crustacea	Pedunculata	Lepadidae	Lepas	*Lepas anatifera anatifera*			+				
Coelenterata	Anthozoa	Actiniaria	Actiniidae	Actinia	*Actinia equina*	+		+	+	+		
Coelenterata	Anthozoa	Actiniaria	Actiniidae	Anthopleura	*Anthopleura xanthogrammica*	+		+			+	

Echinodermata	Echinoidea	Camarodonta	Echinometridae	Anthocidaris	*Anthocidaris crassispina*	+					+	+
Echinodermata	Echinoidea	Camarodonta	Strongylocentrotidae	Hemicentrotus	*Hemicentrotus pulcherrimus*	+					+	
Mollusca	Bivalvia	Mytiloida	Mytilidae	Septifer	*Septifer virgatus*	+	+		+		+	+
Mollusca	Bivalvia	Mytiloida	Mytilidae	Mytilus	*Mytilus galloprovincialis*	+		+	+			+
Mollusca	Bivalvia	Mytiloida	Mytilidae	Lithophaga	*Lithophaga zitteliana*					+		+
Mollusca	Bivalvia	Mytiloida	Mytilidae	Mytilus	*Mytilus coruscus*				+		+	
Mollusca	Bivalvia	Mytiloida	Mytilidae	Modiolus	*Modiolus comptus*						+	+
Mollusca	Bivalvia	Pterioida	Ostreidae	Ostrea							+	

Mollusca	Bivalvia	Veneroida	Glauconomidae	Glauconome	*Glauconome corrugata*				+		+	
Mollusca	Gastropoda	Archaeogastropoda	Trochidae	Chlorostoma	*Chlorostoma rustica*	+	+		+	+	+	+
Mollusca	Gastropoda	Archaeogastropoda	Trochidae	Chlorostoma	*Chlorostoma nigerrima*		+				+	
Mollusca	Gastropoda	Archaeogastropoda	Trochidae	Monodonta	*Monodonta labio*				+		+	
Mollusca	Gastropoda	Archaeogastropoda	Nacellidae	Cellana	*Cellana toreuma*				+	+	+	
Mollusca	Gastropoda	Archaeogastropoda	Calliostomatidae	Calliostoma	*Calliostoma aculeatum*						+	+
Mollusca	Gastropoda	Mesogastropoda	Vermetidae	Serpulorbis	*Serpulorbis imbricata*			+	+		+	+

Mollusca	Gastropoda	Mesogastropoda	Ranellidae	Gyrineum	*Gyrineum natator*						+	
Mollusca	Gastropoda	Neogastropoda	Muricidae	Thais	*Thais clavigera*	+	+	+	+	+	+	+
Mollusca	Gastropoda	Neogastropoda	Buccinidae	Cantharus	*Cantharus cecillei*	+	+	+	+	+	+	+
Mollusca	Gastropoda	Neogastropoda	Turbinidae	Turbo	*Turbo petholatus*	+					+	+
Mollusca	Gastropoda	Neogastropoda	Muricidae	Chicoreus	*Chicoreus asianus*	+					+	+
Mollusca	Gastropoda	Neogastropoda	Muricidae	Thais	*Thais luteostoma*		+	+	+		+	+
Mollusca	Gastropoda	Nudibranchia	Homoiodorididae	Homoiodoris							+	
Mollusca	Polyplacophora	Acanthochitonina	Acanthochitonidae	Acanthochiton	*Acanthochitona rubrolineatus*	+		+	+	+	+	
